# Resonance Raman Probes for Organelle-Specific Labeling in Live Cells

**DOI:** 10.1038/srep28483

**Published:** 2016-06-24

**Authors:** Andrey N. Kuzmin, Artem Pliss, Chang-Keun Lim, Jeongyun Heo, Sehoon Kim, Alexander Rzhevskii, Bobo Gu, Ken-Tye Yong, Shangchun Wen, Paras N. Prasad

**Affiliations:** 1 Institute for Lasers, Photonics and Biophotonics, State University of New York, University at Buffalo, Buffalo, NY 14260, USA; 2 Center for Theragnosis, Korea Institute of Science and Technology, Seoul 136-791, Republic of Korea; 3 Thermo Fisher Scientific, Waltham, MA 02451, USA; 4 Key Laboratory for Micro-/Nano-Optoelectronic Devices of Ministry of Education, School of Physics and Electronics, Hunan University, Changsha 410082, China; 5 School of Electrical and Electronic Engineering, Nanyang Technological University, 639798, Singapore

## Abstract

Raman microspectroscopy provides for high-resolution non-invasive molecular analysis of biological samples and has a breakthrough potential for dissection of cellular molecular composition at a single organelle level. However, the potential of Raman microspectroscopy can be fully realized only when novel types of molecular probes distinguishable in the Raman spectroscopy modality are developed for labeling of specific cellular domains to guide spectrochemical spatial imaging. Here we report on the design of a next generation Raman probe, based on BlackBerry Quencher 650 compound, which provides unprecedentedly high signal intensity through the Resonance Raman (RR) enhancement mechanism. Remarkably, RR enhancement occurs with low-toxic red light, which is close to maximum transparency in the biological optical window. The utility of proposed RR probes was validated for targeting lysosomes in live cultured cells, which enabled identification and subsequent monitoring of dynamic changes in this organelle by Raman imaging.

Raman spectroscopic imaging is among the most powerful tools available for the analysis of molecular organization of cells and tissues. Raman spectroscopy relies on inelastic scattering of incident monochromatic light, wherein the energy of photons is being changed, either by a Stokes or anti-Stokes process, upon interaction with molecules of the sample.

Light scattering on different types of biomolecules generates corresponding bands in the Raman spectra, which enables to resolve certain amino acids, proteins, various classes of lipids and saccharides, as well as DNA and RNA. It is important to note that the intensity of Raman scattering is linearly dependent on the molecular concentrations at the site of spectral measurement, which uniquely allows for quantitative mapping of biomolecular distribution *in situ*^1^,^2^. This valuable feature of Raman spectroscopy has been realized using a Biomolecular Component Analysis (BCA), a powerful algorithm that identifies concentrations of different molecular groups which collectively contribute to the Raman spectrum of the sample^3^. In this regard, Raman spectroscopy has a breakthrough potential for the development of innovatory “omics” technologies (e.g. proteinomics, metabolomics, and lipidomics) at a single-organelle level. An ultimate goal of these research disciplines is a comprehensive characterization and monitoring of biochemical composition in specific cellular organelles, to unravel mechanisms of cellular regulation^4^,^5^. Up to date, most of the data on the molecular composition of subcellular structures have been obtained by mass-spectroscopy. Using this technique, several thousand diverse molecular species have been identified in various cellular compartments^5^,^6^,^7^,^8^. However, molecular profiling with mass-spectroscopy involves cell fractionation and extraction of biomolecules from various organelles, which inherently produces artifacts and is not compatible with live systems^9^. At the same time, conventional live cell imaging techniques, such as fluorescence microscopy, can identify only a few molecular species at a time, which significantly limits their efficiency for comprehensive molecular characterization of single cells and subcellular structures^10^,^11^,^12^,^13^.

In comparison, Raman spectroscopy interrogates all molecules present in the sampling volume of the excitation beam, independently of any extrinsic labels, which is a major strength of this technique. Besides, the Raman signal intensity is not susceptible to photobleaching, thus enabling for long term monitoring of biological samples. Although Raman spectroscopy provides multiple benefits for molecular analysis of a cell, its capabilities as a single-organelle tool are rather limited. A major obstacle of this technique for subcellular analysis is that the location of specific organelles should be resolved to target acquisition of Raman spectra and analyze the content of a single organelle. Meanwhile, subcellular compartments, with a partial exception of mitochondria^1^,^14^, do not exhibit organelle-specific vibrational bands and, therefore, cannot be recognized by the Raman imaging of intrinsic cellular components.

To circumvent this limitation, several research groups, including ours, have applied a bi-modal fluorescence/Raman approach, wherein conventional fluorescence reporters are used to target Raman spectral acquisition to specific cellular compartments^3^,^15^,^16^. However, it became apparent that conventional fluorescence reporters are not well suitable for Raman imaging, mainly due to a strong fluorescence background, masking the Raman signal. Moreover, fluorophores are quickly photobleached at high signal excitation power densities used in the Raman technique, thus making extended monitoring of organelles in live cells impossible^17^.

In parallel to experimentations with fluorescence probes, a first generation of designated Raman reporters has been developed on the basis of deuterium, nitrile or alkyl containing molecular groups, which produce distinctive vibrational bands in the biologically silent region of Raman spectrum^17^,^18^. This advancement, for the first time, enabled detection of specific cellular structures in the Raman modality. However, the signal intensity of the aforementioned Raman tags does not exceed that of native cellular biomolecules, which implies severe limitations in the detection sensitivity. Since, exogenous molecular probes are typically applied at low (nanomolar to micromolar) concentrations to avoid cytotoxicity, the signal intensity from currently existing probes may not be sufficient for detection of labeled organelles in live cells.

Plasmonic enhancement of Raman scattering on metal surfaces, known as Surface Enhanced Raman Spectroscopy (SERS)^19^, has been used to increase signal from Raman probes and improve the detection sensitivity. However, advancing the SERS technology for mapping of intracellular biomolecules is extremely challenging^20^,^21^. First, targeted molecules of interest greatly outnumber plasmonic nanoparticles that can be tolerated by cells without any adverse effect. Second, the size of most biomolecules is incomparably smaller than that of these nanoparticles. Therefore, any intracellular gradient in the distribution of biomolecules cannot be identified by SERS. Besides, bulky metal nanoparticles can disturb activities of biomolecules and produce mechanical damage and other artifacts. Finally, SERS is not applicable in fixed cells, as plasmonic nanoparticles do not penetrate into the cell, even after very extensive permeabilization of cellular membranes. All these factors limit the utility of the SERS technology for intracellular molecular probing.

An optimistic strategy to achieve enhancement of Raman scattering utilizes the Resonance Raman (RR) phenomenon^22^. In this approach, the energy of Raman excitation is adjusted to overlap with an electronic transition of the molecule of interest, such as a Raman reporter, which results in a significant amplification of the light scattering process. This RR technique has been successively applied for biomedical tissue analysis and detection of cancer related abnormalities^23^,^24^. It has also been successfully applied for ultra-sensitive detection of nucleic acids and protein biomolecules. However RR enhancement of cellular biomolecules utilizes highly phototoxic UV light, which is a prohibitive limitation for most live cell studies. At the same time natural pigments, which absorb in visible light, have long been a target RR spectroscopic probing of live cells and tissues^22^. Besides, the advantages of RR and SERS can be combined into an approach known as Surface Enhanced Resonance Raman Spectroscopy (SERRS) when the RR enhancement for some analytes, or reporters, could be accomplished at the excitation wavelength which also excites the plasmonic particles and creates SERS. SERRS was reported to generate unsurpassed enhancement of Raman signal, and has been successfully used for cell-free assays^25^ . However as discussed above, the plasmonic particles have only a limited value for labeling of intracellular molecules. A new concept for Raman molecular probes, which produces unprecedentedly strong Raman signal through RR enhancement and provide capability for intracellular labeling, recently was developed by our group^14^. Our reporters utilized azobenzene (AZO) tags, which were modified to produce RR enhancement under excitation with visible light at 532 nm, which is far less cytotoxic than UV light used in conventional RR spectroscopy. We further synthesized an AZO-RR probe for organelle-specific labeling in live cells and demonstrated its exceptionally high photostability, enabling long term monitoring of the same organelle^14^.

Current Raman microscopy involves fairly long acquisition time, up to several minutes per square micrometer of the sample as well as intense laser illumination (typically 10 mW or higher), and therefore the risk of photodamage to a living specimen has to be carefully considered. In this regards, it has been demonstrated that selecting the excitation wavelengths in the spectral region from the red to the infrared, significantly reduces phototoxicity and enables repeatable spectra acquisition, without compromising functions of the organelle^26^. Therefore, development of RR probes excitable by biologically safe wavelengths will be highly beneficial for Raman microscopy of live samples.

In this paper, we introduce a novel RR molecular recognition probe, designed for identification of organelles or other cellular structures and demonstrate its application by Raman imaging using excitation in a biologically safe wavelength region. A RR reporter based on BlackBerry Quencher 650 (BBQ-650), was developed to produce RR enhancement under excitation in red spectral range, where cellular biomolecules practically do not absorb, thus minimizing phototoxicity. Amplification of Raman signal by the resonance mechanism drastically increases the detection threshold sensitivity as compared to that of conventional spontaneous Raman probes. Besides, this probe produces low fluorescence background that often limits the sensitivity of Raman technique. Using this novel RR reporter, we synthesized a probe for tracking lysosomes in live cells and demonstrate first Raman detection and RR imaging of this type of cellular organelles. An inherent advantage of our approach is that RR imaging can be combined with the mapping of unlabeled cellular macromolecules by spontaneous Raman technique.

## Results and Discussion

### Resonance Enhancement of Raman Signal of BBQ650-NHS

**Figure 1:**
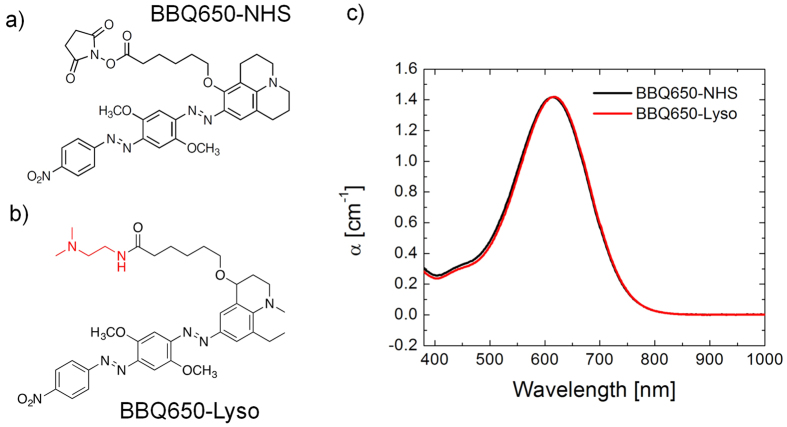
Chemical structure of (**a**) BBQ650-NHS and (**b**) BBQ650-Lyso (lysosome targeting moiety is shown in red). (**c**) Absorption spectrum of 14 μM BBQ650-NHS and BBQ650-Lyso in DMF.

To develop a Raman marker producing resonance signal enhancement upon excitation in the biologically safe spectral region, we selected a commercial BBQ650 quencher. This molecule is composed of the electron accepting nitro group and the electron donating julolidine group coupled to a bis-azobenzene structure (Fig. 1a), which induces a significant red-shift of the electronic absorption spectrum to ~610 nm due to strong intramolecular charge transfer and extended π-conjugation. Figure 1b shows the structure of the RR reporter for tracking lysosomes (BBQ650-Lyso), prepared by tethering BBQ-650 and a lysosome targeting moiety designated here as Lyso. The absorption properties of the BBQ650 and BBQ650-Lyso probes indicate that RR enhancement can be obtained under the red light excitation in the 600–700 nm range (Fig. 1c). Notably, developed as a fluorescence quencher, the BBQ650 probe produces very little fluorescence background, which is an additional valuable benefit of BBQ650 as a Raman probe.

**Figure 2:**
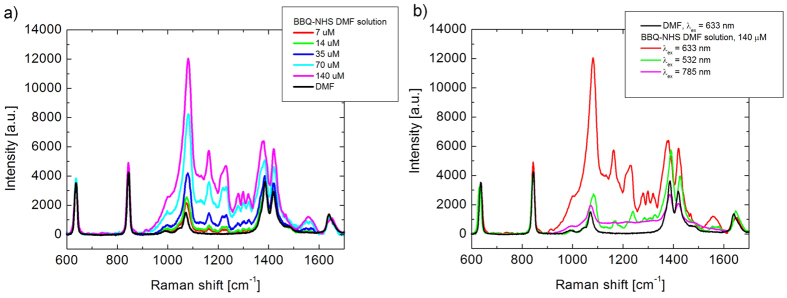
Dependence of the Raman signal of BBQ650-NHS on the excitation wavelength and concentration. (**a**) A correlation between the concentration of BBQ650-NHS solution in DMF and the intensity of Raman spectra under 633 nm excitation. (**b**) Raman spectra of the BBQ-NHS solution in DMF obtained with different excitation sources, as labeled; the band at 1087–1133 cm^−1^ was used for BBQ-NHS detection. It shows that excitation with 633 nm generates a significant RR signal enhancement.

To explore the efficiency of RR enhancement, we have measured the spectra of BBQ650-NHS dissolved in DMF, using the excitation wavelengths of 532, 633 and 785 nm (Fig. 2a). The obtained Raman spectra contained several distinctive bands in the region of 1000–1400 cm^−1^, with the most intense peak at 1087–1133 cm^−1^. Among the tested excitation wavelengths, the highest Raman signal intensity for BBQ650-NHS was obtained under 633 nm excitation (Fig. 2a). This strong Raman signal is most likely resulting from a favorable combination of high extinction coefficient at 633 nm but with a moderate reabsorption of the Raman signal.

At this excitation wavelength, we determined a detection threshold for BBQ650-NHS as 7 μM with a 10 s of signal accumulation time (Fig. 2b), which is two to three orders of magnitude lower than that of common biomolecules, under same experimental measurement conditions^27^. Hence, we concluded a high potential of BBQ650 as a RR probe for cellular bioimaging.

### Design and Synthesis of Resonance Raman Marker

To introduce RR probes for detection of organelles in live cells, we designed a lysosome tracking RR reporter based on BBQ-650 N-hydroxysuccinimide ester (BBQ650-NHS), Fig. 1(a). The lysosome probe (designated as BBQ650-Lyso) was obtained by simple amidation between the NHS ester and the amine modified targeting moiety. As shown in Fig. 1(b), we utilized N,N-dimethyl ethylenediamine for lysosome targeting by permeation and accumulation of the monobasic amine^28^,^29^.

**Figure 3:**
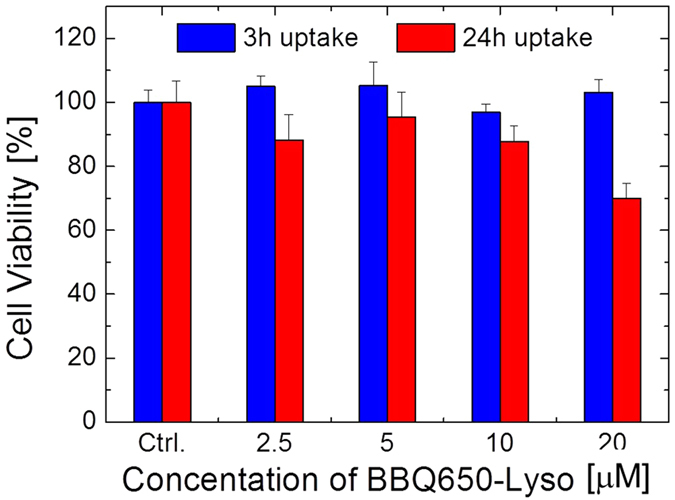
Cytotoxicity of BBQ650-Lyso, evaluated by the MTT colorimetric assay on HeLa cells. As indicated on the plot, cells were treated with 2.5–20 μM of BBQ650-Lyso for 3 h or 24 h. Control (Ctrl) corresponds to untreated cells.

The biocompatibility of the obtained BBQ650-Lyso probe was evaluated by the MTT cytotoxicity assay. In these experiments, cells were incubated with different concentrations of BBQ650-Lyso, ranging from 2.5 μM to 20 μM, for either 3 h or 24 h, and then processed under a standard MTT protocol. As shown in Fig. 3, there was almost no toxic effect for cells following 3 h incubation, for all tested concentrations of the probe. A only significant decrease in the cellular viability was observed, when the probe was used at its highest concentration of 20 μM for 24 h. Considering that organelle labeling in live cells, involves significantly lower concentrations of the probe in the nanomolar to micromolar range, and a shorter incubation time which usually does not exceed one or two hours, we concluded that the BBQ650-Lyso probe is unlikely to cause any measurable cytotoxicity and is well applicable for live cell studies.

**Figure 4:**
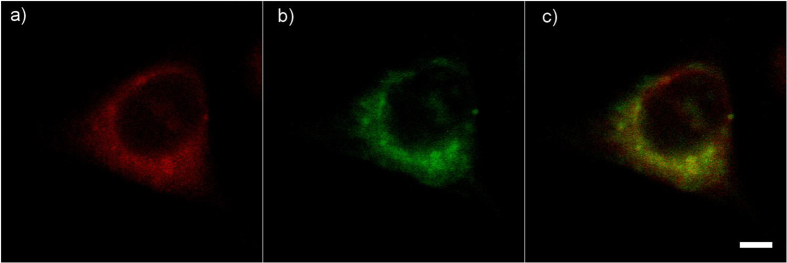
Co-localization of the fluorescence signal from the commercial lysotracker and the Resonance Raman signal from the BBQ650-Lyso probe. The cell was simultaneously stained with the BBQ650-Lyso probe and the commercial lysotracker. Both types of lysosome probes are accumulated in the cytoplasm, and co-localize in the lysosomes, while the nucleus was not stained. Signals from (**a**) commercial fluorescent lysotracker and (**b**) BBQ650-Lyso probe. (**c**) The yellow color on the right panel represents the overlap of the two signals. White bar in (**c**) corresponds to 10 μm. The scanning rate is 4 μs/pixel (full image time scanning ~4 s).

Next, we validated the organelle-targeting specificity of the newly synthesized BBQ650-Lyso probes. Here, live cells were incubated both with BBQ650-Lyso and the commercial fluorescent lysotracker, and the fluorescence and the Raman signal distribution were concurrently acquired (see Materials and Methods). As could be expected from lysotracker, both probes were accumulated in the lysosomes and also were diffusely distributed in the cytoplasm. Our imaging data indicated a significant degree of co-localization of both the fluorescence and the Raman signals in lysosomes, (Fig. 4) which indicates that the BBQ650-Lyso probe synthesized in our study, targets lysosomes with specificity similar to that of commercial probes.

### Raman Spectroscopic imaging of organelles identified by the BBQ650 Resonance Raman Marker

**Figure 5:**
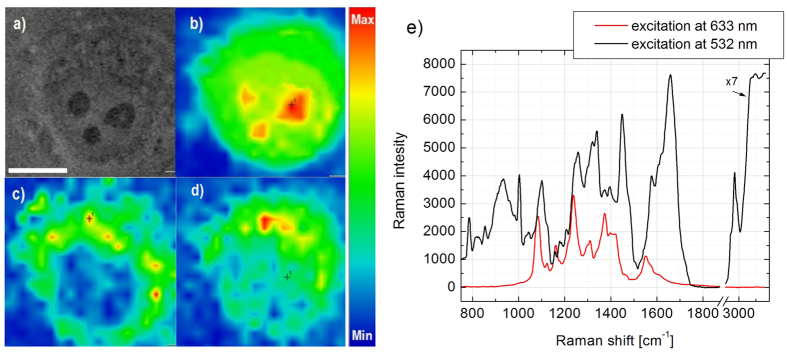
Raman images of HeLa cell incubated with the BBQ-NHS lysosome marker. (**a**) Transmitted light image snap. (**b–d**) Raman images, generated from the spectral peaks assigned to different molecular constituents. (**b**) Spontaneous Raman image under 532 nm excitation corresponding to the proteins 2906–3015 cm^−1^ band. The signal depicts distribution of intracellular proteins in the cell nucleus and cytoplasm. The highest proteins concentration corresponds to the nucleoli (dark domains in (**a**)). (**c**) Resonance Raman image of the BBQ650-Lyso probe (1087–1133 cm^−1^ band) under 633 nm excitation. The BBQ650-Lyso probe accumulated into bright foci in the cytoplasm, consistent with the lysotrackers labeling pattern. (**d**) Spontaneous Raman image for the 1087–1133 cm^−1^ band obtained under 532 nm excitation. The signal is present both in the cell nucleus and the cytoplasm, and does not resemble staining from the lysosome probes. The White bar in (**a**) corresponds to 10 μm. The scanning rates are 2 s/pixel (full image time scanning ~10 min) for (**b,d**) and 0.2 s/pixel (full image time scanning ~1 min) for (**c**). (**e**) Averaged Raman spectra for the entire cell excited by 532 nm (black curve, spontaneous mode, excitation power 10 mW) and BBQ650-Lyso (red curve, resonance mode, excitation power 8 mW). A comparison of the signal intensity at 750–100 cm^−1^ and 1750–400 cm^−1^ demonstrates that under 633 nm excitation by, the signal from cellular macromolecules was at the background level, due to short accumulation time.

Next, we explored the feasibility of simultaneous imaging of BBQ650-based RR probes as well as cellular macromolecules by the spontaneous Raman spectroscopy technique. In these experiments, cells were incubated with the BBQ650-Lyso probe, followed by acquisition of two sequential Raman imaging scans. First, cells were scanned using a 8 mW laser at 633 nm, to produce selective enhancement of the BBQ650-Lyso signal, through the RR mechanism. With a signal accumulation time of 0.2 sec/pixel, an image acquisition of 24 × 24 μm^2^ area corresponding to an average-size cell took about 40 seconds, which satisfies most of live cell imaging tasks. Next, the same cells were scanned with a 532 nm laser at 10 mW excitation power to image the distribution of cellular biomolecules. Despite the higher excitation power, the accumulation time had also to be increased tenfold up to 2 sec/pixel, to obtain a sufficient signal from biomolecules in the absence of resonance enhancement (Fig. 5). The obtained hyper-spectral data were analyzed with the Thermo Scientific™ OMNIC™xi imaging software using embedded image-rendering algorithms. The peak intensities assigned in earlier studies to proteins (2906–3015 cm^−1^)^3^,^30^,^31^, were integrated to generate Raman images (Fig. 5b). Similarly, the signal at 1087–1133 cm^−1^ was processed, for detection of the BBQ650-Lyso probe (Fig. 5c,d) in accordance with the Raman spectrum of this compound (Fig. 3).

We found that owing to RR enhancement, the signal from the BBQ650-Lyso probe at 1087–1133 cm^−1^ was very intense under 633 nm excitation, which enabled for confident imaging of lysosomes in the cell. Raman spectrum of BBQ650-Lyso in lysosomes has the same major peaks at 1080, 1160, 1240 and 1370 cm^−1^, as that of BBQ650-NHS solution. At the same time, in comparison with BBQ650-NHS, where Raman peak centered at 1080 cm^−1^ demonstrated the highest intensity, BBQ650-Lyso does not show this feature. As shown in the integrated Raman spectra the non-resonant Raman signal from the cellular macromolecules was only at the background level due to the short signal accumulation time (Fig. 5e). Contrarily, with the 532 nm excitation, the signal from the BBQ650-Lyso probe was barely detected, despite the prolonged accumulation time. Indeed, mapping the signal of the 1087–1133 cm^−1^ band, does not reveal a similarity between the images obtained under 532 and 633 nm excitations. Most likely, this mismatch can be explained by accumulation of the Raman signal from biomolecular vibrations attributed to C-N and C-C bonds, the tryptophan ring in cellular proteins, and the O-P-O backbone in DNA/RNA^32^,^33^, rather than that of the RR from BBQ650-Lyso, due to the low intracellular concentration of the probe and insignificant levels of RR enhancement with 532 nm excitation.

**Figure 6:**
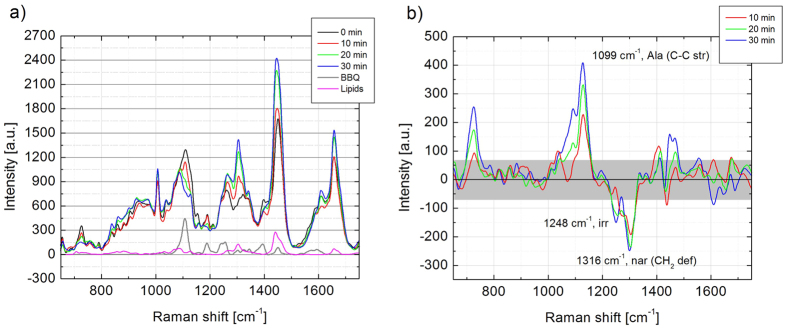
(**a**) Time sequence of the Raman spectra of lysosome, together with the Raman spectra of BBQ (grey curve) and of cellular lipids (magenta curve). (**b**) The residual spectra after subtracting of 10, 20 and 30 min sequential Raman spectra of lysosome from that of 0 min (starting point of measurement). The spectra of BBQ and lipids were subtracted with corresponding weight coefficient as well. Abbreviations: Ala–alanine, str–stretch, irr–irregular, nar–unspecified non-aromatic side-chain, def–deformation. The Grey box shows measurement standard error frame.

Finally, we utilized the RR signal from the BBQ650-Lyso probe to enable time-sequenced acquisition of Raman spectra from a single labeled organelle, and thus validate the utility of this RR probe for selective targeting of subcellular structures. In these experiments, lysosomes in live cells were labeled with the RR probe. The final concentration of the RR probe was ~1 μM, which is within the recommended concentration range for lysotrackers. Labeled organelles were then localized by Raman spectroscopic imaging of cytoplasm and rendering the signal from RR probe. Then, a series of Raman spectra were acquired from single labeled organelles using 633 nm excitation and a longer accumulation time of 120 s for confident characterization of local molecular contents. An intense RR signal from BBQ650 enabled for monitoring of the lysosome position for each spectral measurement. All spectra in the sequence contained the characteristic 1087–1133 cm^−1^ band assigned to BBQ650 (Fig. 6a). As an example of the BCA technique for quantitative monitoring of specific types of molecular vibrations in an organelle, the spectra were processed as follows. First, the spectrum obtained at the first time point was subtracted from the other spectra in the time series. This step enabled to identify changes in the spectra of lysosomes occurring in time. The residual spectra contained prominent contributions of BBQ650 and membrane lipid Raman components. Second, BBQ650 and lipid components were further subtracted from the analyzed spectra. The final residual spectra, obtained after the second subtraction step, are shown in Fig. 6b. We found that during the monitoring time, the intensity of the Raman band centered at 1099 cm^−1^, a common marker of proteins assigned to the alanine C-C-stretch^33^, gradually increased, while another group of protein bands centered at 1248 cm^−1^ and 1316 cm^−1^, which are correspondingly assigned to irregular conformations and deformations of non-aromatic side chains, were decreasing. Such changes reveal that the pool of proteins in lysosome is dynamically changing in time. Thus, the presented here technique can enable studies of biological significance of such transformations in the molecular makeup of a specific organelle.

These data indicate that application of the RR probes may enable rapid location of the organelle of interest in live cells, making it available for monitoring with Raman microspectroscopy. This is a significant step forward in comparison with current bioorthogonal spontaneous Raman markers that cannot be detected at biologically safe concentrations.

## Summary

In this communication, we introduced a next generation of Raman probe for labelling organelles in live cells that utilizes a BBQ650-NHS structure. The proposed probe provides ultrasensitive molecular detection through the mechanism of resonance enhancement of Raman signal. A valuable feature of this probe is that the resonance excitation is achieved using biologically safe light wavelengths in the red region enabling non-invasive live cell imaging. The presented Raman reporter combines all the beneficial features of small molecule probes, such as easy access to dense intracellular structures, absence of unwanted interference with cellular environment, while possessing high detection sensitivity comparable to that of plasmonically enhanced nanoprobes. After specific functionalization, this novel marker can serve as a molecular-selective probe, targeting distinct subcellular domains and making them available for Raman spectroscopic analysis. We have validated this concept for design of RR probes using a BBQ650-Lyso marker for targeting lysosomes in live cultured cells. Using this concept the possibility of dual-mode, resonant and non-resonant, Raman imaging was demonstrated. With this novel technique, the cellular organelles are confidently identified by the signal from the external RR probe using physiologically safe concentration, comparable to that of fluorescence probes. Simultaneously, the conventional Raman imaging of native cellular macromolecules at the site of interest is implemented. Photostability of the RR probe was sufficient for long term tracking of marked lysosomes to perform time-sequential local Raman probing and monitoring the changes of the biomolecular composition in a single organelle of a live cell. This approach will bridge together the advantages of highly versatile molecular recognition probes, currently available only in fluorescence imaging techniques, and a label-free Raman imaging for quantitative biochemical characterization of subcellular structures.

## Materials and Methods

### Preparation of BBQ650-Lyso probe

5 μL of *N,N*-dimethylethylenendiamine was added to 1 mL of 1.4 mM BBQ-650 N-hydroxysuccinimide ester (BBQ650-NHS, Berry & Associate) in dimethylformamide (DMF) at ambient temperature. The reaction mixture was stirred overnight at ambient temperature. The reaction was monitored by TLC (Ethylacetate:Hexane = 1:1). After disappearance of the BBQ650-NHS spot on TLC, the mixture was used for toxicity measurements and cellular imaging experiments without further purifications.

### Cell culture and MTT Assay and labeling of lysosomes

HeLa cells were grown in DMEM supplemented with a 2.5% fetal bovine serum (Gibco), 1% glutamax, 1% Antibiotic Antimycotic Solution (Sigma-Aldrich) at 37 °C in a humidified atmosphere, containing 5% CO_2_. For the MTT Assay, cells were placed in 96-well plates and incubated with the experimental RR lysotracker at concentration ranging from 2.5 to 20 μM for either 3 h or 24 h at 37 °C. Next, cells were washed with PBS and incubated with 0.5 mg/mL of the MTT solution for 3 h, followed by the addition of dimethylsulfoxide. Plates were placed in the microplate spectrophotometer system (Opsys MR, Dynex technologies) and absorbance was recorded at 490 nm. Results were analyzed with the Revelation Quicklink software and are presented as the percentage of the control values.

For staining of lysosomes, HeLa cells were incubated with 1 μM BBQ650-NHS probe and/or 500 nM fluorescent lysotracker (ThermoFisher) for 30 min.

### Raman Single Spectra Measurement and Raman Imaging

Raman spectroscopic measurements were performed by using a confocal Raman microspectrometer based on the SpectraPro 2500i (Acton Research, Trenton, NJ) monochromator equipped with the Spec 10–100B CCD camera (Princeton Instruments, Trenton, NJ) for Raman signal detection .The three laser sources used for excitation are: −532 nm Verdi V-6, 633 nm He-Ne laser (both Coherent, Santa Clara, CA) and 780 nm OPO Levante (APE, Germany) pumped by a High-Q Laser (High Q Laser GmbH, Austria). This configuration enables the measurements within the range of Raman shift of 600–3000 cm^−1^. The spectral resolution for a fixed diffraction grating position (wave number interval of 1210 cm^−1^) was ~1.5 cm^−1^. The Raman spectra of BBQ650 solutions were measured using the following excitation powers: 633 nm–25 mW, 532 nm–25 mW, 785 nm–200 mW. Raman imaging was performed with the DXRxi Raman Imaging Microscope (Thermo Fisher Scientific, Madison, WI). For cell imaging, we used a 60x water immersion objective lens (Olympus, Japan).

The laser power at the sample plane during Raman imaging was 8 mW for 633 nm and 10 mW for 532 nm.

### Co-localization of BBQ650-Lyso probe and commercial fluorescent lysotracker

Live cells where lysosomes were labeled with both BBQ650-Lyso and commercial fluorescent lysotracker, were imaged by the multimodal imaging system described in our earlier publications^3^,^15^. For imaging using fluorescent lysotracker, 10 μW excitation at 532 nm was used in combination with a 60 nm band-pass optical filter centered at 580 nm (FF01-580/60, Semrock, USA). The RR signal of BBQ650-Lyso, generated by 30 mW of 633 nm excitation, was used for imaging, employing a 30 nm band-pass optical filter centered at 687 nm (FF01-687/30, Semrock, USA).

## Additional Information

**How to cite this article**: Kuzmin, A. N. *et al*. Resonance Raman Probes for Organelle-Specific Labeling in Live Cells. *Sci. Rep.* **6**, 28483; doi: 10.1038/srep28483 (2016).

## References

[b1] Schulze H. G., Konorov S. O., Piret J. M., Blades M. W. & Turner R. F. B. Label-free imaging of mammalian cell nucleoli by Raman microspectroscopy. Analyst 138, 3416–3423, doi: 10.1039/C3an00118k (2013). 23636076

[b2] Ferraro J. R. & Nakamoto K. Introductory Raman spectroscopy. (Academic Press, 1994).

[b3] Pliss A., Kuzmin A. N., Kachynski A. V. & Prasad P. N. Nonlinear Optical Imaging and Raman Microspectrometry of the Cell Nucleus throughout the Cell Cycle. Biophys J 99, 3483–3491, doi: 10.1016/j.bpj.2010.06.069 (2010). 21081098 PMC2980749

[b4] Foster L. J. . A mammalian organelle map by protein correlation profiling. Cell 125, 187–199, doi: 10.1016/j.cell.2006.03.022 (2006). 16615899

[b5] Kislinger T. . Global survey of organ and organelle protein expression in mouse: Combined proteomic and transcriptomic profiling. Cell 125, 173–186, doi: 10.1016/j.cell.2006.01.044 (2006). 16615898

[b6] Brunet S. . Organelle proteomics: looking at less to see more. Trends Cell Biol 13, 629–638, doi: 10.1016/j.tcb.2003.10.006 (2003). 14624841

[b7] Ahmad Y., Boisvert F. M., Gregor P., Cobley A. & Lamond A. I. NOPdb: Nucleolar Proteome Database-2008 update. Nucleic Acids Res 37, D181–D184, doi: 10.1093/nar/gkn804 (2009). 18984612 PMC2686505

[b8] Satori C. P. . Bioanalysis of Eukaryotic Organelles. Chemical reviews 113, 2733–2811, doi: 10.1021/cr300354g (2013). 23570618 PMC3676536

[b9] Satori C. P. . Bioanalysis of Eukaryotic Organelles (vol 13, pg 2733, 2013). Chem Rev 113, 5699–5699, doi: 10.1021/cr400254d (2013). PMC3676536 23570618

[b10] Barbe L. . Toward a confocal subcellular atlas of the human proteome. Mol Cell Proteomics 7, 499–508, doi: 10.1074/mcp.M700325-MCP200 (2008). 18029348

[b11] Huh W. K. . Global analysis of protein localization in budding yeast. Nature 425, 686–691, doi: 10.1038/nature02026 (2003). 14562095

[b12] Lippincott-Schwartz J., Snapp E. & Kenworthy A. Studying protein dynamics in living cells. Nat Rev Mol Cell Bio 2, 444–456, doi: 10.1038/35073068 (2001). 11389468

[b13] Fernandez-Gonzalez R., Munoz-Barrutia A., Barcellos-Hoff M. H. & Ortiz-De-Solorzano C. Quantitative *in vivo* microscopy: the return from the ‘omics’. Curr Opin Biotech 17, 501–510, doi: 10.1016/j.copbio.2006.07.005 (2006). 16899361

[b14] Li Y. . Organelle specific imaging in live cells and immuno-labeling using resonance Raman probe. Biomaterials 53, 25–31, doi: 10.1016/j.biomaterials.2015.02.056 (2015). 25890703

[b15] Pliss A., Kuzmin A. N., Kachynski A. V. & Prasad P. N. Biophotonic probing of macromolecular transformations during apoptosis. P Natl Acad Sci USA 107, 12771–12776, doi: 10.1073/pnas.1006374107 (2010). PMC2919951 20615987

[b16] Yadav N. . Transformations of the macromolecular landscape at mitochondria during DNA-damage-induced apoptotic cell death. Cell Death Dis 5, doi: Artn E145310.1038/Cddis.2014.405 (2014). 10.1038/cddis.2014.405 PMC4649512 25299778

[b17] Palonpon A. F., Sodeoka M. & Fujita K. Molecular imaging of live cells by Raman microscopy. Curr Opin Chem Biol 17, 708–715, doi: 10.1016/j.cbpa.2013.05.021 (2013). 23773582

[b18] Yamakoshi H. . Alkyne-Tag Raman Imaging for Visualization of Mobile Small Molecules in Live Cells. Journal of the American Chemical Society 134, 20681–20689, doi: 10.1021/ja308529n (2012). 23198907

[b19] Palonpon A. F. . Raman and SERS microscopy for molecular imaging of live cells. Nat Protoc 8, 677–692, doi: 10.1038/nprot.2013.030 (2013). 23471112

[b20] Hu Q., Tay L. L., Noestheden M. & Pezacki J. P. Mammalian cell surface imaging with nitrile-functionalized nanoprobes: biophysical characterization of aggregation and polarization anisotropy in SERS imaging. J Am Chem Soc 129, 14–15, doi: 10.1021/ja0670005 (2007). 17199265

[b21] Gao F., Zhu Z., Lei J. & Ju H. Raman spectroscopic detection of sub-picomolar DNA by coupling silver catalyzed silver deposition with circular strand-replacement polymerization on magnetic nanoparticles. Chem Commun (Camb) 48, 10603–10605, doi: 10.1039/c2cc35203f (2012). 22930114

[b22] Efremov E. V., Ariese F. & Gooijer C. Achievements in resonance Raman spectroscopy review of a technique with a distinct analytical chemistry potential. Anal Chim Acta 606, 119–134, doi: 10.1016/j.aca.2007.11.006 (2008). 18082644

[b23] Zhou Y. . Human brain cancer studied by resonance Raman spectroscopy. J Biomed Opt 17, 116021, doi: 10.1117/1.JBO.17.11.116021 (2012). 23154776 PMC3499405

[b24] Liu C. H. . Resonance Raman and Raman spectroscopy for breast cancer detection. Technology in cancer research & treatment 12, 371–382, doi: 10.7785/tcrt.2012.500325 (2013). 23448574

[b25] McNay G., Eustace D., Smith W. E., Faulds K. & Graham D. Surface-Enhanced Raman Scattering (SERS) and Surface-Enhanced Resonance Raman Scattering (SERRS): A Review of Applications. Appl Spectrosc 65, 825–837, doi: 10.1366/11-06365 (2011). 21819771

[b26] Pliss A. . Fluctuations and synchrony of RNA synthesis in nucleoli. Integrative biology: quantitative biosciences from nano to macro 7, 681–692, doi: 10.1039/c5ib00008d (2015). 25985251

[b27] Kuzmin A. N., Pliss A. & Prasad P. N. Changes in Biomolecular Profile in a Single Nucleolus during Cell Fixation. Anal Chem, doi: 10.1021/ac503172b (2014). 25268694

[b28] Seglen P. O. & Gordon P. B. Effects of Lysosomotropic Monoamines, Diamines, Amino-Alcohols, and Other Amino-Compounds on Protein-Degradation and Protein-Synthesis in Isolated Rat Hepatocytes. Mol Pharmacol 18, 468–475 (1980). 7464813

[b29] Andrew C. L., Klemm A. R. & Lloyd J. B. Lysosome membrane permeability to amines. Bba-Biomembranes 1330, 71–82, doi: 10.1016/S0005-2736(97)00145-4 (1997). 9375814

[b30] Uzunbajakava N. . Nonresonant Raman imaging of protein distribution in single human cells. Biopolymers 72, 1–9, doi: 10.1002/bip.10246 (2003). 12400086

[b31] Zinin P. V. . Visible, near-infrared, and ultraviolet laser-excited Raman spectroscopy of the monocytes/macrophages (U937) cells. J Raman Spectrosc 41, 268–274, doi: 10.1002/jrs.2444 (2010).

[b32] Duguid J., Bloomfield V. A., Benevides J. & Thomas G. J. Raman Spectral Studies of Nucleic-Acids 44. Raman-Spectroscopy of DNA-Metal Complexes .1. Interactions and Conformational Effects of the Divalent-Cations-Mg, Ca, Sr, Ba, Mn, Co, Ni, Cu, Pd, and Cd. Biophys J 65, 1916–1928 (1993). 8298021 10.1016/S0006-3495(93)81263-3 PMC1225927

[b33] Raso S. W., Clark P. L., Haase-Pettingell C., King J. & Thomas G. J. Distinct cysteine sulfhydryl environments detected by analysis of Raman S-H markers of Cys ->Ser mutant proteins. J Mol Biol 307, 899–911, doi: 10.1006/jmbi.2001.4476 (2001). 11273709

