# Corrigendum: Resonance Raman Probes for Organelle-Specific Labeling in Live Cells

**DOI:** 10.1038/srep32469

**Published:** 2016-09-02

**Authors:** Andrey N. Kuzmin, Artem Pliss, Chang-Keun Lim, Jeongyun Heo, Sehoon Kim, Alexander Rzhevskii, Bobo Gu, Ken-Tye Yong, Shuangchun Wen, Paras N. Prasad

Scientific Reports 6: Article number: 2848310.1038/srep28483; published online: 06 24 2016; updated: 09 02 2016

The original version of this Article contained an error in the spelling of the author Shuangchun Wen, which was incorrectly given as Shangchun Wen. This error has now been corrected in the PDF and HTML versions of the Article.

